# Peribulbar anesthesia for cataract surgery: Effect of lidocaine warming and alkalinization on injection pain, motor and sensory nerve blockade

**DOI:** 10.4103/0301-4738.60072

**Published:** 2010

**Authors:** Venkatakrishnan Jaichandran, Lingam Vijaya, Ronnie Jacob George, Bhanulakshmi InderMohan

**Affiliations:** Department of Anaesthesiology, Vision Research Foundation, Sankara Nethralaya, 41/18, College Road, Nungambakkam, Chennai - 600 006, Tamil Nadu, India; 1 Department of Glaucoma, Vision Research Foundation, Sankara Nethralaya, 41/18, College Road, Nungambakkam, Chennai - 600 006, Tamil Nadu, India

**Keywords:** Hyaluronidase, lidocaine, peribulbar injection

## Abstract

**Aim:**

To compare self-reported pain and efficacy of warmed, alkalinized, and warmed alkalinized lidocaine with plain 2% lidocaine at room temperature for peribulbar anesthesia in cataract surgery.

**Materials and Methods:**

Through a prospective, single-blinded, randomized, controlled clinical trial 200 patients were divided into four groups. They received either lidocaine at operating room temperature 18°C, control group (Group C), lidocaine warmed to 37°C (Group W), lidocaine alkalinized to a pH of 7.09 ± 0.10 (Group B) or lidocaine at 37°C alkalinized to a pH of 6.94 ± 0.05 (Group WB). All solutions contained Inj. Hyaluronidase 50 IU/ml. Pain was assessed using a 10-cm visual analog score scale. Time of onset of sensory and motor blockade and time to onset of postoperative pain were recorded by a blinded observer.

**Results:**

Mean pain score was significantly lower in Group B and WB compared with Group C (*P* < 0.001). Onset of analgesia was delayed in Group C compared with Group B (*P* = 0.021) and WB (*P* < 0.001). Mean time taken for the onset of complete akinesia and supplementation required for the block was significantly lower in Group B. Time of onset of pain after operation was significantly earlier in Group W compared with Group C (*P* = 0.036).

**Conclusion:**

Alkalinized lidocaine with or without warming produced less pain than lidocaine injected at room temperature. Alkalinization enhances the effect of warming for sensory nerve blockade, but warming does not enhance alkalinization, in fact it reduces the efficacy of alkalinized solution for blocking the motor nerves in the eye.

Pain during injection of local anesthetic solution is common and this is partly explained by the direct tissue irritation caused by injecting an acidic solution, Lidocaine hydrochloride (L-HCL).[1] The increase in relative concentration of the non-ionized form allows for a more rapid diffusion through the tissues and might result in almost immediate sensory nerve blockade.[2–4] The nociceptor receptors are also less sensitive to the non-ionized form of the drug.[3] Hence pain perceived is less during injection of either warmed[5–7] or alkalinized[8–10] solutions as they contain increased non-ionized fraction of the drug form.

Both alkalinization and warming have been found to produce synergistic effects in intradermal anesthesia.[11,12] There is very little information on the synergistic effects of alkalinization and warming in peribulbar anesthesia. Hence we undertook this prospective, single-blinded, randomized controlled clinical trial to determine whether alkalinization and warming possess synergistic effects in peribulbar anesthesia. Our main aim of the study was to compare the pain perception occurring during injection of warmed, alkalinized, and warmed alkalinized lidocaine with 2% lidocaine solution at room temperature. We also wanted to determine the efficacy of the solutions by comparing the time of onset of sensory and motor nerve blockade between them.

## Materials and Methods

### Preliminary *in vitro* workup

The mean pH + SD of 10 ml of 2% lidocaine solution (Xylocaine 2%, AstraZeneca, Bangalore, India) *n* = 6, with Hyaluronidase 50 IU/ml (Hynidase, Shreya Life Sciences, Aurangabad, India), was found to be 6.52 ± 0.08 (range: 6.39-6.59). pH was measured using a digital pH meter (model LI 120; ELICO Hyderabad, India). For alkalizing the above solution, 0.5 ml of preservative-free 7.5% sodium bicarbonate was required. The mean pH ± SD of the alkalinized lidocaine solution (*n* = 6), was 7.09 ± 0.10 (range: 7.00-7.23). The mean time interval needed for warming 2% lidocaine solution (*n* = 6) to a temperature of 40°C was 6 min 54 sec ± 17 sec. For standardization, lidocaine vials were kept in a water bath (Labserve) set at 40°C for 10 min. The mean time interval recorded for lidocaine solution to attain a temperature of 37°C from 40° was 120 ± 24.49 sec. Hence to ensure that the temperature of the solution is around 37°C during injection into the peribulbar space, the warmed solution had to be injected within 2 min from the removal of the solution from the water bath. For alkalizing warmed lidocaine solution, 0.25 ml of 7.5% sodium bicarbonate was needed and used. The mean pH ± SD of warmed and alkalinized lidocaine solution (*n* = 6) was 6.94 ± 0.05 (6.90-7.00). If the warmed lidocaine solution was alkalinized beyond the above range, precipitation of the solution occurred.

After obtaining approval from the Institutional Review Board of the Vision Research Foundation (Chennai, India), 200 patients gave written informed consent for this study. All patients were aged 40 years and above and were scheduled for phacoemulsification cataract surgery under local anesthesia. Patients with history of previous intraocular surgery under local anesthesia, known allergy to lidocaine, mental retardation, one-eyed patients and those with inadequate vision to appreciate the visual analog scale (less than 20/200 on Snellen visual acuity chart) were excluded. Two patients refused to participate in the study and one patient was excluded because conventional extracapsular extraction was performed.

No preoperative sedatives were administered.

All eligible patients were randomized into one of the four groups to receive a peribulbar injection from any one of the following solutions:

1. Group (Gr) C: 10 ml of plain 2% lidocaine solution at room temperature, 18°C (Control group)
2. Gr W: 10 ml of 2% lidocaine solution at 37°C
3. Gr B: 10 ml of 2% lidocaine solution buffered to an estimated pH of 7.09 ± 0.10
4. Gr WB: 10 ml of 2% lidocaine solution at 37°C buffered to an estimated pH of 6.94 ± 0.05

Randomization was done based on a computer-generated random table. Injection hyaluronidase (50 IU ml^−1^) was added prior to alkalizing or warming, to all anesthetic solutions.

Routine monitoring for all patients included electro cardiogram (ECG), noninvasive arterial pressure monitoring and pulse oximetery. Patients were clearly explained about the procedure involved in the peribulbar block and also about the use of visual analog scale (VAS) of 10 cm to evaluate the pain perceived by them, zero cm representing no pain and 10 cm representing the most severe pain.

To maintain the uniformity of the technique, peribulbar block was administered by a single non-blinded anesthetist, experienced in ophthalmic anesthesia, and the same blinded surgeon performed the surgery for all the patients. The block was administered using a 23-G, 1” blunt steel needle. The needle was first inserted through the lid at a point between the lateral third and medial two-thirds of the lower orbital margin, with the bevel facing the globe. It was then advanced in a superomedial direction (parallel to the plane of the orbital floor), for a distance of approximately 25 mm to the equator of the globe, where the anesthetic solution was injected, outside the muscle cone at a rate of 5 ml in 10 sec.[13] Immediately after the injection, the VAS was shown to the subjects to mark the pain perceived by them during the injection. Patients were requested not to take into consideration the pain caused by the needle prick, but only that provoked during injection of the solution in the periocular space.

The globe was then compressed gently for 2 min with the middle three fingers placed over a sterile gauze pad on the upper eye lid with the middle finger pressing directly down on the eyeball. Two minutes following the first injection, the second injection was administered in the superomedial compartment. The needle was introduced through the upper lid at about 2 mm medial and inferior to the supraorbital notch. It was then advanced in a sagittal plane under the roof of the orbit for a maximal depth of 25 mm where the remaining 5 ml of local anesthetic was injected at a similar rate as given for the inferior injection.[13] Digital compression of the globe was again performed as described above.

The efficacy of the block was evaluated by a second blinded anesthetist every 30 sec after administration of the superior injection. Analgesic onset was assessed by holding the bulbar conjunctiva both medially and laterally with toothed forceps. Adequacy of akinesia was determined by the absence of ocular movements (< 1 mm) in all directions. Supplemental injections with the same anesthetic mixture were given at 5 min of interval following the superior injection in case of residual movement (>1 mm). If there was superior and/or medial movement, the superior injection with 1 to 2 ml of injectate was repeated. Similarly, inferior injection with 1 to 2 ml of injectate was given if there was any inferior and/or lateral movement.

Vital signs were monitored throughout the surgery. Patients were encouraged to communicate with the surgeon regarding pain during surgery and if required sub Tenon's supplementation was given with 2 ml of plain lidocaine by the surgeon. At the end of the surgery the efficacy of anesthesia was graded by the surgeon, blinded to the solution used, based on the adequacy of akinesia and anesthesia throughout the procedure and the need for intraoperative supplementation (Annex 1). The presence of pain in the first 24 h after operation and the need for any oral analgesic, Tab. Paracetamol were also recorded.

**Annex 1:** Grading of efficacy of peribulbar block

Grade	Efficacy of anesthesia
5	Adequate anesthesia and akinesia throughout surgery without supplementation
4	Adequate anesthesia, inadequate akinesia, no supplementation
3	Inadequate anesthesia, adequate akinesia, supplementation required
2	Inadequate akinesia, adequate anesthesia, supplementation required
1	Inadequate akinesia and anesthesia, supplementation required
0	Inadequate anesthesia or akinesia or any other complication, necessitating termination of the operative procedure, despite supplementation

The sample size was calculated to detect a significant difference of 2 in VAS score with power of the study 80% and α equal to 0.05.

All continuous variables are presented by Mean±SD and it was analyzed by Student's t-test. The categorical datas were presented by frequency with percentages and it was analyzed by Chi-square test. One-way ANOVA with Dunnett test was used for comparison between the groups. Results were considered significant if *P* was < 0.05. SPSS 13 software package was used for statistical analysis.

## Results

The groups were similar in age, gender, body weight and duration of operation [Table 1]. The pain score, time of onset of analgesia and akinesia are enumerated in Table 2. Mean pain score was significantly higher in Gr C compared with Gr W (*P* = 0.002), Gr B (*P* < 0.001) and WB (*P* < 0.001). Mean time of onset of analgesia was delayed in Gr C compared with Gr B (*P* = 0.021) and WB (*P* < 0.001). The difference between Gr C and W in sensory blockade was not significant (*P* = 0.579). Onset of motor nerve blockade was earlier in Gr B compared with Gr C (*P* = 0.033), W (*P* < 0.001) and WB (*P* = 0.038). At 5 min of interval following superior injection, significant number of patients in Gr W (54%) and WB (48%) required supplementation of the block once compared with Gr B (24%) (*P* = 0.002 for Gr W and *P* = 0.012 for Gr WB), Table 2.

**Table 1:** Patient characteristics in the four groups

	Group C (*n* = 50)	Group W (*n* = 50)	Group B (*n* = 50)	Group WB (*n* = 50)
Age (years)	62.6 ± 8.9	59.5 ± 10.3	63.3 ± 10.5	59.78 ± 9.3
Sex (Male/Female)	27/23	33/17	28/22	35/15
Body weight (Kg)	68.4 ± 12.7	65.9 ± 11.7	67.8 ± 13.8	67.3 ± 11.91
Operation time (min)	19.4 ± 3.4	20.6 ± 4.3	20.8 ± 4.4	19.3 ± 3.1

Data are mean ± SD. None of the differences were significant; *P* > 0.05

**Table 2:** Mean pain score obtained and time taken for sensory and motor nerve blockade

Variable	Group C	Group W	Group B	Group WB
Pain Score	2.71 ± 1.93	1.68 ± 1.47	1.13 ± 1.15*	1.11 ± 1.22*
95% CI	2.16-3.26	1.26-2.09	0.80-1.46	0.76-1.46
Onset of analgesia (min)	2.59 ± 0.28	2.54 ± 0.35	2.45 ± 0.25†	2.34 ± 1.01*
95% CI	2.52-2.68	2.44-2.64	2.38-2.52	2.31-2.37
Onset of akinesia (min)	5.46 ± 2.89	6.06 ± 3.04	4.06 ± 2.31‡	5.07 ± 2.75
95% CI	4.64-6.28	5.20-6.93	3.40-4.72	4.30-5.86
Supplementation needed (%)	18 (36)	27 (54)§	12 (24)	24 (48)‖

Data are mean ± SD; CI - confidence interval;

* *P* < 0.001 compared with control;

† *P* = 0.021 compared with control;

‡ *P* = 0.033 compared with control;

§ P=0.002 Gr W compared with Gr B and

‖ P=0.012 Gr WB compared with GrB

Adequate anesthesia and akinesia throughout surgery was achieved in all cases of Gr B and WB. Time of onset of pain after operation was earlier in Gr W compared with Gr C (*P* = 0.036) [Table 3].

**Table 3:** Postoperative pain (0-24 h)

	Group C	Group W	Group B	Group WB
Postoperative pain	15 (30)	18 (36)	13 (26)	17 (34)
Need for oral analgesics	9 (18)	10 (20)	8 (16)	10 (20)
Time of onset of pain, min	193.33 ± 150.57	75.00 ± 86.25*	116.15 ± 136.03	99.71 ± 101.03
95% CI	109.95-276.71	32.11-117.89	33.95-198.36	47.76-151.65

Data are number (%); CI - Confidence interval;

* *P* = 0.036 compared with control

## Discussion

Local anesthetics are weak bases. To improve their stability they are supplied in acidic solution Lidocaine hydrochloride (L-HCL).[14] In this form, local anesthetics exist mainly in the ionized fraction. Based on the Hendersson-Hasselbach equation, i.e. the ratio between ionized and non-ionized species being a function of both the pKa (dissociation constant, lidocaine = 7.80) of the drug and the pH of the dissolving medium, the addition of sodium bicarbonate to L-HCL, increases the non-ionized form of the drug.[15]

The pKa value is also temperature dependent. Hence as local anesthetic is warmed, the pKa value decreases (pKa for lidocaine is 7.57 at 40°C)[16] and the proportion of uncharged drug available for action increases.[17–20] This non-ionized lipophilic form of the drug apart from producing a rapid sensory nerve blockade also helps in reducing the amount of pain perceived by patients during injection as the nociceptor receptors are less sensitive to this form of the drug.[3] Hence, similar to the previous studies, in our study too we found that pain was significantly reduced during injection into the periocular space either with pre-warmed local anesthetic[5–7] or alkalinized lidocaine.[8–10] Injecting lidocaine at room temperature (18°C) resulted in significant pain subjectively as it produced higher mean pain scores.

Theoretically speaking, both warming and alkalinization of lidocaine should produce lowest pain scores for injection. But in our study we found that alkalinization with or without warming lidocaine produced lowest mean pain score. Thus it is quite evident that, for this iatrogenic pain reduction no synergistic effect exists between warming and alkalinization of lidocaine.

Apart from a reduction in the pain perception, warmed solution did not help to achieve early analgesia or akinesia in the eye. Krause *et al*. found that there is no significant difference in bulbar analgesia and akinesia after retrobulbar anesthesia between injections of warm and cold anesthetic solutions.[21] Injecting warmed and alkalinized solution produced earlier onset of analgesia compared with room temperature lidocaine but this effect was not noted with warmed solution alone. The above findings suggest the fact that alkalinization enhances the effect of warming in blocking the sensory nerves early. Interestingly, akinesia of the globe was achieved significantly earlier with alkalinized lidocaine solution but not with warmed alkalinized lidocaine solution. Warming was found to reduce the efficacy of alkalinized solution for motor nerve blockade.

During surgery, one patient (2.0%) each in Gr C and W required subtenon's supplementation due to inadequate anesthesia and two patients (4.0%) in Gr W required subtenon's supplementation due to inadequate akinesia and anesthesia. The time of onset of postoperative pain was found to be significantly earlier in patients injected with warmed than room temperature lidocaine solution.

Even though both alkalinization and warming are known to increase the non-ionized active form of the drug,[15–19] the increase in the efficacy of the block and early sensory and motor nerve blockade occurred only with alkalinized lidocaine solution. This can be explained partly by the presence of hyaluronidase in the anesthetic mixture. Hyaluronidase depolymerizes hyaluronidic acid, leading to liquefaction of the gelatinous interstitial barrier, preventing compartmentalization from occurring and thus theoretically promoting the spread of local anesthetic.[22] Previous studies have demonstrated that pH-adjustment of the local anesthetic mixture improved the activity of hyaluronidase.[22–24] A pH range of 6.4-7.4 was found to be the optimal pH range for hyaluronidase activity.[24] This however, does not explain the delay in the onset time for extraocular muscle blockade in warmed alkalinized lidocaine solution (mean pH of 6.94 ± 0.05 ranging from 6.90-7.00).

The only limitation encountered in the study was that the anesthesiologist who performed the block was non-blinded, since his fingers were in contact with the syringe, and he could feel the temperature change and infer the group to which the patient belonged. To minimize bias a second blinded anesthesiologist evaluated the time of onset of analgesia and akinesia and decided on the need for supplemental injections if required. Variations in block and surgical technique were reduced to the minimum as only a single anesthesiologist administered injections and the same surgeon performed all cataract surgeries.

## Conclusion

Unlike in intradermal anesthesia alkalinization and warming do not possess a synergistic effect in peribulbar anesthesia for iatrogenic pain reduction occurring during injection of lidocaine. Also, we found that alkalinization enhances the effect of warming for blocking the sensory nerves, but warming does not enhance alkalinization and actually reduces the efficacy of alkalinized solution for blocking the motor nerves in the eye.

Alkalinization of lidocaine is the best choice for patients undergoing cataract surgery under periocular anesthesia as it produced the least painful injection, achieved early analgesia and akinesia with fewer supplemental injections.
